# Bovine Satellite Cells Isolated after 2 and 5 Days of Tissue Storage Maintain the Proliferative and Myogenic Capacity Needed for Cultured Meat Production

**DOI:** 10.3390/ijms22168376

**Published:** 2021-08-04

**Authors:** Stig Skrivergaard, Martin Krøyer Rasmussen, Margrethe Therkildsen, Jette Feveile Young

**Affiliations:** Department of Food Science, Aarhus University, Agro Food Park 48, 8200 Aarhus, Denmark; stsk@food.au.dk (S.S.); martink.rasmussen@food.au.dk (M.K.R.); jettef.young@food.au.dk (J.F.Y.)

**Keywords:** cultured meat, bovine satellite cells, myogenic potential, cell proliferation, tissue storage, primary cell isolation

## Abstract

Cultured meat is an emerging alternative food technology which aims to deliver a more ethical, sustainable, and healthy muscle-tissue-derived food item compared to conventional meat. As start-up companies are rapidly forming and accelerating this technology, many aspects of this multi-faceted science have still not been investigated in academia. In this study, we investigated if bovine satellite cells with the ability to proliferate and undergo myogenic differentiation could be isolated after extended tissue storage, for the purpose of increasing the practicality for cultured meat production. Proliferation of bovine satellite cells isolated on the day of arrival or after 2 and 5 days of tissue storage were analyzed by metabolic and DNA-based assays, while their myogenic characteristics were investigated using RT-qPCR and immunofluorescence. Extended tissue storage up to 5 days did not negatively affect proliferation nor the ability to undergo fusion and create myosin heavy chain-positive myotubes. The expression patterns of myogenic and muscle-specific genes were also not affected after tissue storage. In fact, the data indicated a positive trend in terms of myogenic potential after tissue storage, although it was non-significant. These results suggest that the timeframe of which viable myogenic satellite cells can be isolated and used for cultured meat production can be greatly extended by proper tissue storage.

## 1. Introduction

Cultured meat or in vitro meat production is a new concept which could have enormous impact on our meat production systems and the overall impact from meat production on the climate [1,2]. Since the first public reveal of a cultivated piece of meat by Marc Post in 2013 [3], the arena of cultured meat has expanded considerably. Several small-scale industrial start-up companies, attracting increasing amounts of investments, are now focused on delivering the first genuine cultured meat product to the consumers, based on bovine, porcine, avian and pesci cells [4]. However, from an academic perspective this scientific field is very much in its infancy and the knowledge regarding the complex biology and intricate biotechnical techniques needed for such a production system is scarce. The interplay between many research fields, such as stem cell isolation and characterization, bioreactor design and cell culture scale-up, growth media optimization, three-dimensional scaffolds and sensory and nutritional evaluations [1,5,6] does indeed complicate cultured meat research. However, this interdisciplinary approach is necessary for the successful development of a sustainable, nutritional and tasty in vitro meat product.

Cultured meat can be achieved by cultivating animal cells with a high proliferative capacity, which have the ability to differentiate into mature muscle fibers, i.e., the main component of traditional meat. Several stem cell types can theoretically be utilized for this purpose, e.g., embryonic stem cells (ESCs) [7,8], mesenchymal stem cells (MSCs) [9,10,11] and induced pluripotent stem cells (iPSCs) [12], however the most reliable and well-studied cell source at this time are the satellite cells (SCs) [5].

Satellite cells are adult stem cells located between the sarcolemma and the basal lamina of skeletal muscle fibers, typically being in a quiescent state until activation by, e.g., muscle damage, which sets in motion several myogenic regulatory factors (MRFs) initiating the proliferation, differentiation and fusion of new multinucleated muscle cells. This innate ability to repair damaged muscle tissue is the reason why SCs are a great candidate for creating a cell-based meat product. Intricate cell biology determines the SC fate, as it differentiates through the myoblast, myocyte and myotube phases, of which extensive review articles already exists [13,14]. The expression of canonical myogenic transcription factors and specific muscle proteins can be used to follow this development of myotubes and hence the formation of muscle tissue. The most well-characterized and important SC transcription factors in this differentiation pathway are the Pax7, MyoD1, Myf5, Myf6, Mef2 and myogenin proteins [13,14,15,16]. The PAX7 (paired box protein 7) gene is widely used as an identifier of pre-differentiated SCs [14], while the MYOD1 (myoblast determination protein 1) [17,18], MEF2A (myocyte-specific enhancer factor 2A) [19,20,21] and MYF5 (myogenic factor 5) [16,22] genes are encoding transcription factors important for early activation and myogenesis. The MYF6 gene (myogenic factor 6, also known as MRF4) is also activated in the early phase of myogenesis, however expression is maintained throughout maturation of adult skeletal muscle [15,23]. The last myogenic regulatory factor (MRF), myogenin (MYOG gene), is essential for terminal differentiation [16]. Muscle-specific proteins such as desmin and myosin heavy chain (MHC) are important for the contractile function of myofibers [24] and are well established as markers for terminal differentiation and maturation of myotubes [5]. Desmin is a very abundant muscle-specific intermediate filament protein [25], while the myosin heavy chain (MHC) protein is the main motor protein in the myofibrils necessary for muscle contraction and is encoded by the MYH gene [26]. These muscle-proteins can be utilized in immunofluorescence imaging to evaluate the myogenic potential of the satellite cells after allowing them to differentiate and undergo fusion. The heme-protein myoglobin (MB gene) is not a canonical SC differentiation marker, however it might be important in future cultured meat production as it might improve proliferation, color and taste of cultivated muscle cells [6,27]. Therefore, including this muscle-protein in eventual cultured meat studies could further elucidate its role in SC biology and myofiber formation.

The animal stem cells can be obtained from a muscle biopsy from live animals or sampled at slaughter. In a purely theoretical context, this means that a large cultured meat production could be sustained without animal slaughter as well as significantly reducing the numbers of animals required for the global meat production [28,29], although it might require an impractical number of bioreactors of current size to feed a city of 5 million people [30].

One important aspect of this task is the immediate logistics of the muscle biopsies and the SC isolations. Common practice and earlier studies have dictated the importance of the immediate isolation of primary cells, as extended storage highly effects the viability of these cells [31,32,33]. However, if tissue storage could extend the cell isolation timeframe, it would improve practicality and effective cell yield when receiving larger amounts of tissue. In this study, we analyzed bovine SCs isolated from muscle tissue, either immediately upon arrival (approximately 2 h after sacrificing the animal and dissecting the muscle) or after two and five days of storage at 4 °C. We analyzed the growth characteristics of the isolated satellite cells, the expression profiles of important muscle-specific markers and the ability of the cells to differentiate and fuse into mature myotubes.

## 2. Result

### 2.1. Proliferation of Bovine Satellite Cells Isolated from Fresh and Stored Muscle Tissue

**Figure 1:**
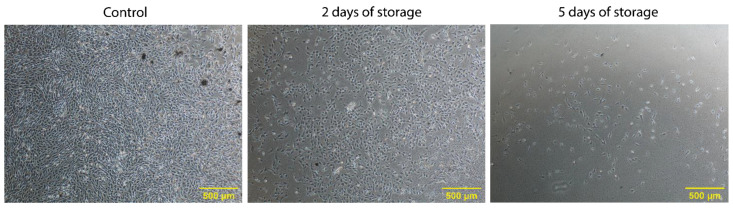
Representative phase contrast images of isolated cells after 6 days of cultivation. Cells isolated from fresh tissue (control) and after 2 and 5 days of storage. The images were obtained 6 days after thawing the cryopreserved isolates. Scale bar = 500 µm at 10× magnification.

After isolation of bovine satellite cells from the *M. semimembranosus* from three Holstein dairy cows either on the day of slaughter (Control) or after 2, 5 and 8 days of tissue storage, cells were cryopreserved. Initially, when the cryopreserved isolates were thawed and cultivated, viable cells could be observed in the samples from stored tissue, even after 5 days of storage (Figure 1). However, it was also evident that the tissue storage had a negative effect on the number of viable cells and their initial lag phase. Figure 1 shows this effect after 6 days of cultivation, which resulted in one additional day for the “2 days of storage” sample to reach confluence similar to the control and four additional days for the “5 days of storage” sample. This negative effect of especially 5 days of storage was seen across all three cows. In addition, a high biological variance in initial cell numbers between cows was observed (data not shown). The isolates after 8 days of storage showed very few viable cells and were omitted from further testing.

The cells used in this study were cultivated from early-attaching cells, i.e., cells attaching within two days in growth media. The growth media removed after two days contained late-attaching cells, which were also capable of further proliferation indicating a greater potential from the total cell isolate than the one elaborated on in this manuscript (Supplementary Figure S1A). Even the tissue left over from the isolation procedure showed potential, as it was possible to grow cells from this using the tissue explant technique (Supplementary Figure S1B).

**Figure 2:**
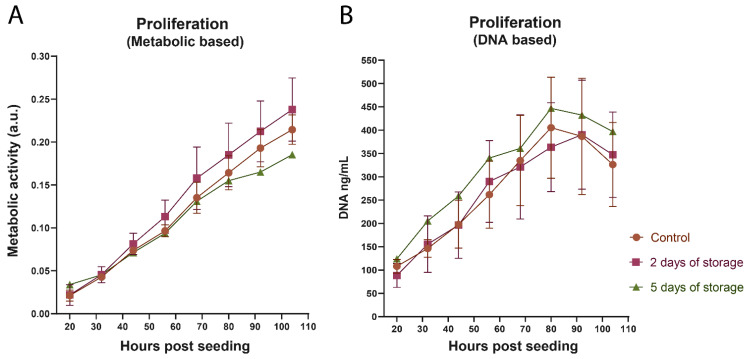
Proliferation of isolated cells measured by metabolic activity (**A**) and DNA concentrations (**B**). Satellite cells isolated on the day of tissue arrival (control) and after 2 and 5 days of storage were analyzed measuring metabolic activity (Figure 2A) and DNA concentration (Figure 2B). The data points represent the mean ±SEM based on three cows in triplicates (*n* = 3) for control and 2 days of storage, while 5 days of storage is only based on one cow in triplicates (*n* = 1). The Y-axis in Figure 2A is metabolic activity in arbitrary units (a.u.) based on 450 nm absorption. The Y-axis in Figure 2B is DNA concentration in ng/mL based on a known internal DNA standard.

After this initial cultivation phase, the proliferation of the isolated bovine satellite cells was quantitatively measured during a 20–104 h period after cell seeding (Figure 2). The cells used for these experiments had only been passaged once prior to cell seeding. The measured data from “5 days of storage” only included cells isolated from one cow due to the initial lag phase and reduced cell numbers. The proliferation assays indicated healthy growing cell populations across all three isolation times, based on both metabolic activity (Figure 2A) and the amount of cellular DNA (Figure 2B). Interestingly, no significant differences were found between the fresh control samples and the stored samples in this time range. This indicated that tissue storage had no effect on cell proliferation after the initial cultivation period.

The isolates were also assessed in a long-term culture, which extended beyond 40 days with regular passages (every 3–4 days) (Supplementary Figure S2A). All isolates showed viable cells throughout the entirety of the experiment, however the cumulative population doubling levels (PDL) varied greatly between isolates, which was probably due to a shift in the cell population type indicated by changed morphology (Supplementary Figure S2B).

### 2.2. Fusion Potential of Bovine Satellite Cells Isolated from Fresh and Stored Muscle Tissue

**Figure 3:**
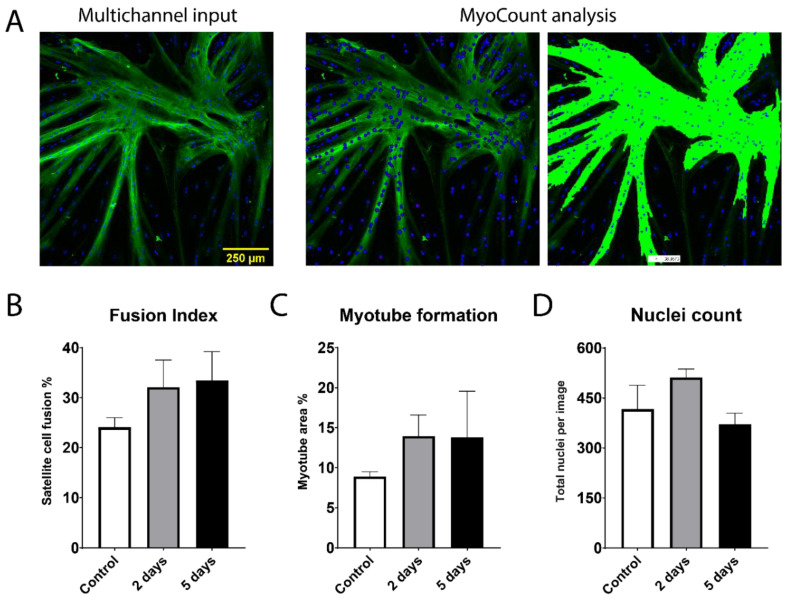
Satellite cell fusion and formation of MHC-positive myotubes. (**A**) Multichannel fluorescence images of myosin heavy chain (MHC) (green channel) and Hoechst-stained nuclei (blue channel) were overlayed before MyoCount analysis. Fusion index (**B**), myotube formation (**C**) and total nuclei count (**D**) were quantified. The three groups compared are the freshly isolated samples (Control) and the 2 and 5 days of storage isolates. Data is presented as mean ± SEM based on three cows with 2 × 4 images each (*n* = 3).

Apart from proliferation and expansion of cells, also the ability to differentiate, fuse and create myofibers are recognized as an important parameter in the production of cultured meat. If the highly structured texture of meat is to be replicated in cultured meat, it is necessary to retain the cellular ability to undergo fusion and create long well-aligned proteinaceous myofibers. Therefore, we analyzed the fusion potential of the three groups of satellite cell isolations (control, 2 and 5 days of storage) using myosin heavy chain (MHC), as a myotube marker protein, and MyoCount image processing (Figure 3A). This analysis quantified the Fusion Index, the myotube coverage and the number of nuclei after 3 days of fusion. All cell samples across cow origin and storage time were able to differentiate and create MHC-positive myotubes. Interestingly, this analysis showed that SCs isolated after 2 and 5 days of storage had slightly higher average fusion potential than the non-stored control samples (although non-significant), both in terms of fusion index and myotube formation (Figure 3B,C).

The fusion index after 2 days of storage (~32%) and 5 days of storage (~35%) was higher when compared to the control cells (~24%) (Figure 3B). The myotube formation was increased from ~9% in the control samples to ~14% after 2 days of storage and to ~15% after 5 days of storage (Figure 3C). In the total nuclei count, the 2 days of storage samples (512 nuclei average) scored higher than both control (417 nuclei average) and 5 days of storage (384 nuclei average) (Figure 3D) (although non-significant).

This experimental setup for fusion analysis was also performed on the late-attaching cells from the supernatant mentioned earlier, which was capable of myotube formation, albeit in a slightly lower degree compared to the main isolates analyzed here (data not shown). The cells from the long-term culture were also analyzed (after more than 40 days with continuous passaging), however no fusion was observed at this point.

### 2.3. Myogenic and Muscle-Specific mRNA Expression Profiles of Bovine Satellite Cells Isolated from Fresh and Stored Muscle Tissue

**Figure 4:**
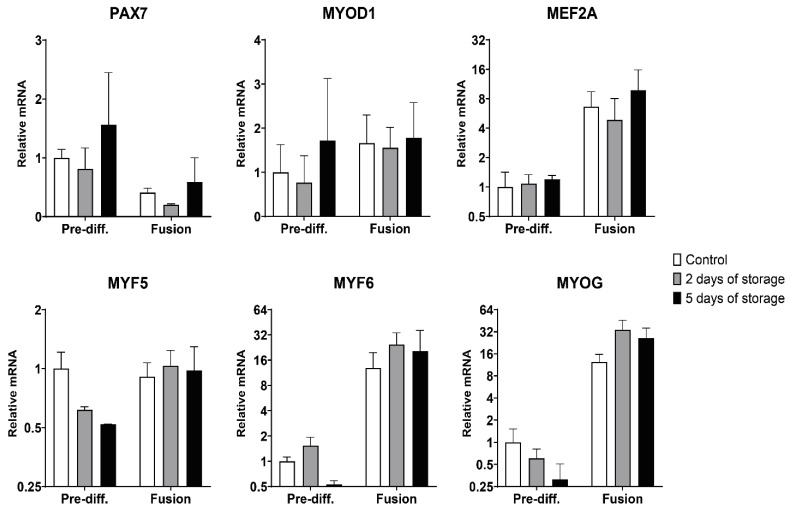
Quantitative RT-PCR analysis of myogenic gene expression before and after fusion. Pre-diff.: pre-differentiated. Total RNA was isolated from pre-differentiated (Pre-diff.) satellite cells and after they had undergone fusion, which was used in a two-step reverse transcriptase-qPCR (RT-qPCR) to quantify mRNA expression of the specific myogenic transcription factors: PAX7 (paired box 7), MYOD1 (myogenic differentiation 1), MEF2A (myocyte enhancer factor 2A), MYF5 (myogenic factor 5), MYF6 (myogenic factor 6) and MYOG (myogenin). The data is presented as relative mRNA levels (relative to pre-differentiated control samples). The Y-axis is log2 scaled. The data is presented as mean ± SEM based on three cows (*n* = 3), except for the 5 days of storage data which is from only two cows (*n* = 2).

To further characterize the satellite cells isolated and their ability to undergo myogenic differentiation, we analyzed the expression of important myogenic markers and muscle-specific genes using quantitative RT-PCR. These analyses were performed on pre-differentiated cells harvested from passage 1 and cells that were seeded and allowed to differentiate and fuse. The canonical myogenic transcriptional markers investigated included the four myogenic regulatory factors MYF5, MYOD1, MYF6 and MYOG genes, as well as the PAX7 and MEF2A genes (Figure 4). The PAX7 gene showed similar expression across all three isolation groups with a decreased expression after fusion in all three groups (Figure 4), as would be expected. The MYOD1 expression was not changed after the fusion protocol and no significant differences was observed between control and stored samples. MEF2A expression was upregulated ~7-fold, ~5-fold and ~10-fold in the control samples and the 2 and 5 days of storage samples, respectively. However, there were no significant differences between the three isolation times neither before nor after fusion. The MYF5 gene expression was not changed after fusion (relative to pre-differentiated control), but a trend of lower expression in the pre-differentiated satellite cells isolated after 2 days of storage (0.62-fold) and after 5 days of storage (0.52-fold) when compared to the control cells was observed (Figure 4). MYF6 is activated in the early phase of myogenesis (as MYOD1 and MYF5), however as expression is maintained throughout maturation the relative mRNA values were highly elevated after fusion, being increased ~13-fold, ~25-fold and ~21-fold in the control, 2 days and 5 days of storage samples, respectively. The expression after fusion was not significantly different between the three isolation times.

The last myogenic regulatory factor, myogenin (MYOG gene), being essential for terminal differentiation was highly upregulated after fusion as expected (Figure 4), with ~12-fold (control), ~34-fold (2 days of storage) and ~26-fold (5 days of storage) increases. These relative fold inductions after fusion were higher after 2 days of storage and 5 days of storage when compared to the control, even though their initial pre-differentiation expression levels showed a reduced trend (although with non-significant differences).

**Figure 5:**
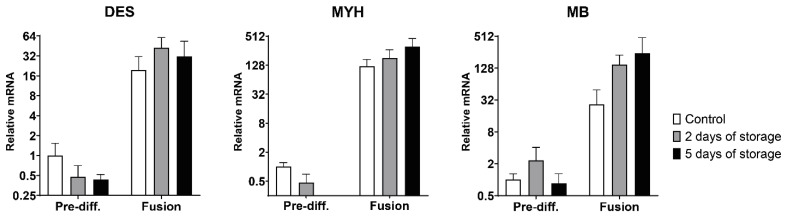
Quantitative RT-PCR analysis of muscle-specific gene expression before and after fusion. Relative mRNA expression of the muscle-specific genes: DES (desmin), MYH (myosin heavy chain) and MB (myoglobin) in pre-differentiated (Pre-diff.) satellite cells and after fusion. The data is presented as relative mRNA levels (relative to pre-differentiated control samples). The Y-axis is log2 scaled. The data is presented as mean ±SEM based on three cows (*n* = 3), except for the 5 days of storage data which is from only two cows (*n* = 2). MYH Pre-diff. data from 5 days of storage was omitted as it was only detected in one cow.

After terminal differentiation and fusion, the muscle-specific proteins desmin and myosin heavy chain should be abundantly expressed in maturating myotubes. We therefore investigated the expression of these genes (Figure 5). The desmin (DES) gene showed a high upregulation after fusion in the control samples (~20-fold increase), as well as in the 2 days of storage (~42-fold increase) and 5 days of storage samples (~32-fold increase) compared to the pre-differentiated control. No significant differences were observed between the three isolation times. The myosin heavy chain (MHC) protein already utilized in the fusion potential experiments is encoded by the MYH gene, which in this case was drastically upregulated after the fusion protocol (Figure 5). The control cells increased expression by an average of ~122-fold, while the cells isolated after 2 and 5 days of storage increased expression by ~181-fold and ~314-fold, respectively. In the pre-differentiated state, the MYH expression was reduced by 0.47-fold after 2 days of storage, while being omitted in the 5 days of storage isolation group as it was only detected in one biological replicate. We also investigated the expression of the myoglobin gene (MB), which was also very highly upregulated after fusion in a range similar to myosin heavy chain, with a ~27-fold increase in control cells and higher increases after 2 days of storage (~150-fold) and 5 days of storage (~243-fold) (Figure 5). In general, a high biological variance was observed in the gene expression analyses, especially after fusion when concerning the many-fold upregulated genes (Supplementary Table S2).

The collagen type alpha-1 gene (COL1A1) was analyzed to reveal the presence of any fibroblastic tendencies of the cell population, however no significant changes were observed (Supplementary Figure S3). Cells from the long-term culture experiment were investigated and compared to the early-passage cells used above. In this comparison, it was observed that the long-term cells had drastically reduced expression of several myogenic genes (Supplementary Figure S4).

## 3. Discussion

The science of cultured meat is a new and trending field of research which incorporates several complex biological and technical elements, such as cell isolation and characterization, bioreactor design and cell culture scale-up, growth media optimization, three-dimensional scaffolds and sensory and nutritional evaluations [1,5,6]. However, the available literature on this is limited, positioning the science of cultured meat very much in its infancy. We have in this study focused on the initial part of the many complex processes needed for the successful production of cultured meat. The immediate logistics involved with the biopsy handling and satellite cell isolation was the focus in our study, as we analyzed the performance of cell isolates from either fresh muscle-tissue or after 2 and 5 days of storage, for the purpose of extending and optimizing the isolation strategy.

Generally, when isolating primary cells from tissue, time is of the essence, as extended storage time severely impacts cell viability [31,32,33]. This negative effect was initially observed when starting our satellite cell cultures. Satellite cells isolated from fresh tissue were in higher numbers after the first 7–10 days, compared to the stored samples, especially the isolates after 5 days of storage performed poorly in this phase, while 8 days of storage resulted in almost no viable cells. However, after this initial cultivation phase, the early proliferation of isolates after 2 and 5 days of storage was comparable to the fresh control isolates, with no significant differences, based on both metabolic activity and DNA concentration. Similar results were also observed in the preliminary studies, in which no negative effects of storage time were observed in proliferation assays or in long-term culture. Other studies have observed viable cells after extended tissue storage. Isolation of viable murine and human satellite cells have been achieved up to 14 and 17 days post-mortem, respectively, albeit with a much lower count than freshly isolated [34], and as an extreme example, fibroblast-like cells have been isolated from goat skin tissue after 160 days post-mortem [35].

Interestingly, in the same satellite cell study by Latil et al. [34], they found a decrease in the myogenic markers, MyoD1 and myogenin, a decrease in oxygen consumption and an increase in ROS production in cells isolated 4 and 8 days post-mortem. The decrease in myogenin (MYOG) expression after storage was also noticed in our pre-differentiated cells after storage, although this was only a trend with no statistical significance. On the contrary, after fusion the myogenin expression was increased with storage time in our experiments, albeit not significant, which unfortunately was not investigated in their study. In terms of fusion potential, it was interesting to see in our study that the isolates from stored tissue consistently performed better than the control samples. The satellite cells isolated after 2 and 5 days of storage were both able to achieve a higher fusion index and myotube formation. Furthermore, these satellite cells from stored tissue had similar myogenic expression patterns as the non-stored control samples, with highly upregulated genes after differentiation and fusion. The tissue storage even led to increased myogenin, myosin heavy chain and myoglobin expression after fusion. These results are somewhat conflicting with the observations made by Latil and colleagues [34], however, their findings regarding oxygen consumption and ROS production might hint at the mechanism in which our satellite cells were better primed for differentiation after tissue storage.

Oxygen levels are certainly important physical parameters in stem cell culture and in satellite cell myogenesis [36,37]. Anoxia has been observed to increase both proliferation and viability of neural stem cells, while glucose deprivation was highly detrimental [38]. Furthermore, hypoxia has been observed to upregulate MRFs, proliferation rate, self-renewal capacity and determine satellite cell fate [39,40]. This could perhaps explain some of the observation made in our study, as extended tissue storage inevitably would lead to depleted oxygen levels. However, at a certain point this positive effect will surely be reversed, as we observed for 5 and 8 days of storage with much reduced cell numbers, perhaps also being affected by the deprivation of glucose levels in the muscle tissue.

The obtained results also indicated that robust and myogenic satellite cell cultures could be isolated without the need of an additional purification step (e.g., Percoll gradient or FACS), which has been observed previously in isolations from bovine, porcine, equine and avian muscle tissue [41]. Nevertheless, as our long-term culture experiments showed a gradual change of cell population morphology, as well as decreased expression of myogenic genes and loss of fusion potential, the purity of the isolated SCs might be of great importance for long-term cultivation. Maintaining the myogenic potential throughout large-scale bioreactor expansion could be problematic, however evidence suggest that the “stemness” of SCs can be maintained through external factors [42,43]. Furthermore, the tissue explant technique was a very simple method of achieving more viable and myogenic cells from the same tissue. These cells were not characterized in detail, but visible fusion was observed in several instances and murine myoblasts of high purity have previously been isolated using this with the addition of a pre-plating step [44]. The high biological variance between the samples from the three cows was very evident, and several factors might attribute to these, one being the exact location of the muscle tissue sampling as satellite cell content might vary depending on fiber type and proximity to capillaries [14].

The establishment of myoblast cell cultures from frozen muscle tissue has also been successfully performed, without any reported negative effects [45], thereby making long term storage possible, although the size of tissue and method of cryopreservation should be carefully considered [46]. Depending on future production schemes and practices, extending tissue storage for several days in refrigerated media might be preferred. Our results indicated that isolation of satellite cells after tissue storage for up to 5 days was possible without negatively affecting proliferation and myogenic potential. Interestingly, this extended storage might even be beneficial for differentiation and fusion if a lower amount of starting material can be negated. However, the decrease in initial cell numbers with increasing storage time must be carefully considered before extending individual cell isolation procedures. Nevertheless, this study indicates that the timeframe in which viable myogenic satellite cells can be isolated from muscle tissue can be greatly extended by cold storage, a result that may open the potential of sourcing satellite cells from slaughter animals in a future circular bio-resource perspective.

## 4. Materials and Methods

### 4.1. Chemicals

All chemicals used for cell culture work such as DPBS, trypsin and media components were from Gibco™ (Thermo Scientific, Rockford, IL, USA), except gentamicin, penicillin and streptomycin antibiotics which were from Sigma (Saint Louis, MO, USA). All other chemicals used were from Sigma unless otherwise stated.

### 4.2. Satellite Cell Isolation

Directly after the sacrifice of three Holstein dairy cows (1327, 1519 and 1113 days old) at a commercial slaughterhouse (Danish Crown, Aalborg, Denmark), the *M. semimembranosus* was removed and transported on ice to the cell laboratory at Department of Food Science, Aarhus University within 2 h. Biopsies were taken from each muscle, approximately at the center, with a custom-made autoclavable metal tube (18 cm long, 2 cm diameter) with a 3D printed handle. Biopsies were either used immediately for isolation or stored at 4 °C for 2, 5 or 8 days in a 50 mL falcon tube containing DMEM (61965-026, Gibco) supplemented with: gentamicin 0.2 mg/mL, penicillin 200 units/mL and streptomycin 0.2 mg/mL, amphotericin B 5 µg/mL. Satellite cells were isolated by finely mincing the muscle tissue, in DPBS with 1% (*w/v*) D-glucose and antibiotics (gentamicin 0.2 mg/mL, penicillin 200 units/mL and streptomycin 0.2 mg/mL, amphotericin B 5 µg/mL), with a small sterile scissor. Then 5 g of minced tissue was subjected to 1 h of enzyme digest at 37 °C in a 50 mL tube using 20 mL of digest media (0.25% (*v/v*) trypsin, 427.5 U/mL collagenase type II (Worthington-Biochem, Lakewood, NJ, USA), 0.01% DNase and 1% (*w/v*) D-glucose in DPBS (without Ca^2+^/Mg^2+^)). The tubes were inverted every 5 mins and trituration were performed after 30 mins of digest using a 25 mL serological pipette. After trituration the tubes were centrifuged for 10 s at 100× g and the supernatant was collected in a new 50 mL tube with ice cold growth media consisting of DMEM (61965-026, Gibco) with 10% FBS, 10% HS, 1mM sodium pyruvate and 1× antibiotics (gentamicin 0.1 mg/mL, penicillin 100 units/mL and streptomycin 0.1 mg/mL, amphotericin B 2.5 µg/mL. This first cell pool was pelleted at 1000× *g* for 10 mins at 4 °C and resuspended in ice-cold growth media and left on ice. Another 20 mL of pre-warmed digest media was added to the muscle tissue and after an additional 30 mins of digest, the trituration was repeated, and the second cell suspension was pooled together with the first. The cell suspensions were then, in succession, filtered through 100 μm and 40 μm cell strainers (Corning, Bedford, MA, USA). The obtained cell suspensions were centrifuged for 10 mins at 1000× *g* at 4 °C and resuspended in 5 mL of ice-cold growth media with 10% (*v/v*) DMSO before being cryopreserved in five cryovials with approximately 1 mL in each vial.

### 4.3. Cell Culture and Population Doubling Level (PDL)

Cell culture vessels were coated with Matrigel Matrix (Corning) diluted 1:50 in phenol red-free DMEM by incubating for 1 h followed washing with DPBS. Cryopreserved cell samples were quickly thawed and diluted in growth media, before being pelleted at 500× *g* for 10 mins at 4 °C and resuspended in 37 °C growth media to remove DMSO-containing freeze media. Satellite cells were initially cultured in 12-well plates using growth media at 37 °C in a 5% CO_2_ humidified atmosphere before cell experiments. For the long-term cultures, cells were grown in 6-well plates and passaged every 3–4 days using 0.25% trypsin in DPBS (without Ca^2+^/Mg^2+^) for cell detachment. Cells were counted using a Burker–Turk hemacytometer, 30,000 cells were seeded per well (3125 cells/cm^2^) and the PDL was calculated using the equation:

PDL = 3.32 × (logC_i_ − logC_f_) + PDL_0_ (1)

C_i_ = the initial number of cells being seeded. C_f_ = the final number of cells after culture. PDL_0_ = the initial cell population doubling level.

### 4.4. WST-1 and PicoGreen Assays

Satellite cells were seeded at a density of 1000 cells per well (3125 cells/cm^2^) in Matrigel coated 96-well plates in 100 μL growth media. After 20 h WST-1 reagent (Roche) was added (10 μL per well) and incubated for 2 h before absorbance at 450 nm was measured (600 nm reference wavelength was used). After WST-1 measurement, the wells were washed with DPBS and cells were lysed by adding 50 μL RIPA lysis buffer per well and incubating for 5 mins at 37 °C. Then 50 μL of TE buffer was added and mixed, before 50 μL of the lysate was transferred to a black 96-well plate and stored at −20 °C. The WST-1 and lysis procedures were performed every 12 h for 4 days. The lysate was analyzed by mixing with 50 μL PicoGreen reagent (Thermo Scientific) (working concentration as defined by manufacturer), incubating for 15 mins at room temperature, and measuring the fluorescence at 485 nm excitation and 528 nm emission using a BioTek (Winooski, VT, USA) plate reader. The DNA standard included in the PicoGreen assay was diluted in RIPA:TE buffer (1:1).

### 4.5. Fluorescence Microscopy

Satellite cells were seeded at a density of 5000 cells/well in a Matrigel coated Ibidi μ-slide (8-well) (Gräfelfing, Germany). Cells were allowed to proliferate for 3 days in 300 μL growth media before differentiation and fusion was initiated by 300 μL fusion media (growth media with only 5% FBS and without HS supplements) with a 3-day incubation. In the preliminary study, 2000 cells/well were allowed to proliferate and differentiate in growth media for 7 days without media change. After myotube formation, the cells were fixed by 3.7% PFA for 10 mins and permeabilized by 0.1% Triton-X for 15 mins. After washing cells were incubated in a 1% (*w/v*) BSA DPBS-Tween 20 (0.1% *v/v*) blocking solution for 1 h at room temperature. Primary anti-myosin heavy chain antibody (M4276, Sigma) diluted 1:400 in DPBS-Tween 20 (0.1% *v/v*) with 0.1% BSA was added (200 μL/well) and incubated overnight at 4 °C. Cells were washed three times with DPBS-Tween 20 before secondary anti-mouse Alexa Fluor 488 antibody (SAB4600388, Sigma) at 1:400 was incubated for 2 h at room temperature. In the last 30 mins Hoechst 33342 (Thermo Scientific) was added in a 1:1000 concentration. Cells were washed three times with DPBS-Tween 20 before covered in protective mounting media (20 mM Tris-HCl pH 8, 0.5% N-propyl gallate, 90% glycerol). Images were taken using a Nikon Eclipse (Tokyo, Japan) confocal microscope. The appropriate laser and gain settings was adjusted based on a negative control sample without primary antibody to eliminate background fluorescence from unspecific binding. Four images were taken from each duplicate well for all samples. Images were processed using ImageJ software before being quantified using MyoCount [47], in which Fusion Index (% nuclei within MHC-positive myotubes, with at least 3 nuclei), myotube formation (% image area coverage of myotubes) and nuclei count (total nuclei per image) was obtained.

### 4.6. Quantitative RT-PCR

Satellite cells were analyzed in their undifferentiated state before seeding as well as after differentiation and fusion. Satellite cells were seeded in Matrigel coated 12-well plates (8000 cells/well) and grown for 3 days in growth media before changing to fusion media for 3 days. In the preliminary study, 4000 cells/well were allowed to proliferate and differentiate in growth media for 7 days without media change. Total RNA was isolated from two duplicate wells and pooled using Trizol reagent according to the manufacture’s guidelines. Reverse transcriptase was performed on 200 ng total RNA template using the SuperScript cDNA kit (Thermo Scientific) according to the manufacturer’s protocol. The cDNA was diluted 1:5 with RNase-free water before q-PCR analysis in technical triplicates was performed using TaqMan Universal PCR Master Mix (Thermo Scientific) according to previously described protocol [48]. Specific primer and TaqMan probes (Supplementary Table S1) were designed using species specific gene sequences (Ensemble gene) as described elsewhere [49] and custom-made (LGC Biosearch Technologies, Risskov, Denmark). The NormFinder algorithm-based software [50] was used to determine the best suitable reference gene amongst three previously validated candidates; RPLP0 [51,52,53], TBP [51,54,55] and UTX [52,56,57]. The comparative 2^−(ΔΔCt)^ method was utilized for graphical representation with all numbers relative to the pre-differentiated state of the non-stored control samples.

### 4.7. Statistical Analysis

GraphPad Prism 9 software (La Jolla, CA, USA) was utilized for statistical analysis. Two-tailed unpaired t-test was used to determine statistical significance between stored samples versus control samples with *p* < 0.05. All graphical data was presented as the mean value ± SEM using GraphPad Prism.

## Supplementary Materials

The Supplementary Materials are available online at https://www.mdpi.com/article/10.3390/ijms22168376/s1.
