# Supplementary material for: Bovine Satellite Cells Isolated after 2 and 5 Days of Tissue Storage Maintain the Proliferative and Myogenic Capacity Needed for Cultured Meat Production
Source: Int J Mol Sci. 2021 Aug 4;22(16):8376. doi: 10.3390/ijms22168376 (PMC8395070; doi:10.3390/ijms22168376)
Supplement: Supplementary file 1 [file ijms-22-08376-s001.zip › ijms-1290984-supplementary.pdf]

Supplementary data:

Table S1: Primer and probe sequences used in RT-qPCR

Sequences are listed as 5'-3' and probes are all of the TAMRA type except three which are minor groove binding (MGB) probes.

Gene	Forward primer	Reverse primer	Probe
COL1A1	AGGAAAAGAGGCACGTCTGGTA	CCCTGGCCGCCATACTC	CCGGCGGATTCCAGTT (MGB)
DES	GAGTACCGCCACCAGATCCA	TCCCTCATCTGCCTCATCAGT	TCCTACACCTGCGAGATCGACGCC
MB	TGCCAAGCATCCTTCAGACTT	CCATGTCATTCCGGAACAGTT	ATGCCCAGGCTGCCATGAGCAA
MEF2A	GCAGGGCCTGGTGTACTCA	GCATTCCTGGCGAGTTGAAG	CCAACTGCCTACAACACAGACTACTCGCTG
MYF5	ACCAGCCCCACCTCAAGTT	TGGACCAGATAGGGCTGTTACA	CTCTGATGGCATGCCT (MGB)
MYF6	GGAGGTGGTGGAGAAGTAACTCA	GCAGGGAGGGTGGGATCTT	TCCGGACGTTCTCCACGGAGCA
MYH	GCCCCGCCCCACAT	CGTTTCGTGTTCACAGTCTTTCC	TCTCCATCTCTGACAACGCCTATCAGTTCA
MYOD1	GGCCTTCGAGACGCTCAA	ATGGCGTTGCGCAGGAT	CGCTGCACGTCTAGCAACCCAAACC
MYOG	CCCTACAGACGCCCACAATC	AGCGACATCCTCCACTGTGAT	CACTCCCTCACCTCCATCGTGGACA
PAX7	CCGCCTCCAATCACATGAA	GGGTTGCTCAGAATGCTCATC	TCAGCAACGGCCTGTCGCCTC
RPLP0	AACCCGGCTCTGGAGAAACT	TGAACACGAAGCCCACATTC	TTGCCTCACATCCGG (MGB)
TBP	AAGCGTTTTGCTGCTGTAATCA	CCATCTTCCCAGAACTGAATATCA	AGAATAAGAGAGCCCCGCACCACTGC
UXT	GATTTGGGCTGTAACTTCTTCGTT	ACCATATCCAAGGGCCACATAG	ACACAGTGGTCCCAGACACTTCACGGA

Table S2: Raw data from quantitative RT-PCR analysis of muscle-specific gene expression before and after fusion

Relative mRNA expression of all investigated myogenic genes from the three individual cows before and after fusion. Mean and SEM (standard error of the mean) is calculated from the data in each row (biological replicates). N.d. (not detected).

PAX7

		Cow 1	Cow 2	Cow 3	Mean	SEM
Control	Pre-diff.	1,119	0,715	1,166	1,000	0,143
Control	Fusion	0,320	0,558	0,351	0,410	0,075
2 days	Pre-diff.	0,357	1,513	0,562	0,811	0,356
2 days	Fusion	0,197	0,230	0,183	0,203	0,014
5 days	Pre-diff.	2,446		0,683	1,565	0,882
5 days	Fusion	0,999		0,175	0,587	0,412

MYF5

		Cow 1	Cow 2	Cow 3	Mean	SEM
Control	Pre-diff.	1,155	1,267	0,578	1,000	0,214
Control	Fusion	0,704	0,798	1,228	0,910	0,161
2 days	Pre-diff.	0,654	0,577	0,617	0,616	0,022
2 days	Fusion	1,059	0,667	1,376	1,034	0,205
5 days	Pre-diff.	0,517		0,520	0,519	0,001
5 days	Fusion	0,661		1,295	0,978	0,317

MYOD1

		Cow 1	Cow 2	Cow 3	Mean	SEM
Control	Pre-diff.	2,252	0,385	0,363	1,000	0,626
Control	Fusion	2,922	1,194	0,872	1,663	0,636
2 days	Pre-diff.	1,987	0,084	0,224	0,765	0,612
2 days	Fusion	1,710	2,270	0,686	1,555	0,464
5 days	Pre-diff.	3,126		0,315	1,720	1,405
5 days	Fusion	2,581		0,989	1,785	0,796

MYF6

		Cow 1	Cow 2	Cow 3	Mean	SEM
Control	Pre-diff.	1,244	0,888	0,868	1,000	0,122
Control	Fusion	26,275	8,246	4,120	12,881	6,802
2 days	Pre-diff.	0,823	1,557	2,223	1,534	0,404
2 days	Fusion	42,714	19,418	11,462	24,531	9,377
5 days	Pre-diff.	0,585		0,471	0,528	0,057
5 days	Fusion	36,126		4,838	20,482	15,644

MEF2A

		Cow 1	Cow 2	Cow 3	Mean	SEM
Control	Pre-diff.	1,786	0,349	0,865	1,000	0,420
Control	Fusion	3,594	4,030	12,268	6,630	2,821
2 days	Pre-diff.	1,511	1,109	0,629	1,083	0,255
2 days	Fusion	2,174	1,206	11,192	4,858	3,180
5 days	Pre-diff.	1,314		1,090	1,202	0,112
5 days	Fusion	3,857		15,765	9,811	5,954

MYOG

		Cow 1	Cow 2	Cow 3	Mean	SEM
Control	Pre-diff.	1,933	0,129	0,938	1,000	0,522
Control	Fusion	17,362	5,941	13,813	12,372	3,375
2 days	Pre-diff.	0,886	0,204	0,723	0,604	0,205
2 days	Fusion	31,457	55,949	14,884	34,097	11,928
5 days	Pre-diff.	0,120		0,507	0,314	0,193
5 days	Fusion	35,891		16,336	26,114	9,777

DES

		Cow 1	Cow 2	Cow 3	Mean	SEM
Control	Pre-diff.	1,923	0,107	0,970	1,000	0,524
Control	Fusion	42,592	11,090	5,294	19,659	11,588
2 days	Pre-diff.	0,852	0,064	0,509	0,475	0,228
2 days	Fusion	50,818	69,077	7,006	42,300	18,418
5 days	Pre-diff.	0,352		0,516	0,434	0,082
5 days	Fusion	53,238		9,951	31,594	21,643

MYH

		Cow 1	Cow 2	Cow 3	Mean	SEM
Control	Pre-diff.	0,786	n.d.	1,214	1,000	0,214
Control	Fusion	181,908	30,920	152,352	121,727	46,198
2 days	Pre-diff.	0,238	n.d.	0,700	0,469	0,231
2 days	Fusion	344,142	49,814	148,023	180,660	86,518
5 days	Pre-diff.	n.d.		n.d.		
5 days	Fusion	168,730		459,149	313,940	145,210

MB

		Cow 1	Cow 2	Cow 3	Mean	SEM
Control	Pre-diff.	1,192	1,366	0,441	1,000	0,284
Control	Fusion	72,985	3,718	2,708	26,470	23,259
2 days	Pre-diff.	0,440	5,843	0,705	2,329	1,759
2 days	Fusion	217,161	231,021	2,165	150,116	74,083
5 days	Pre-diff.	1,296		0,420	0,858	0,438
5 days	Fusion	481,442		5,410	243,426	238,016

COL1A1

		Cow 1	Cow 2	Cow 3	Mean	SEM
Control	Pre-diff.	0,807	0,138	2,055	1,000	0,562
Control	Fusion	1,005	1,264	4,214	2,161	1,029
2 days	Pre-diff.	0,228	0,287	3,910	1,475	1,218
2 days	Fusion	1,140	2,276	6,838	3,418	1,741
5 days	Pre-diff.	1,043		1,896	1,470	0,426
5 days	Fusion	1,906		4,591	3,248	1,342

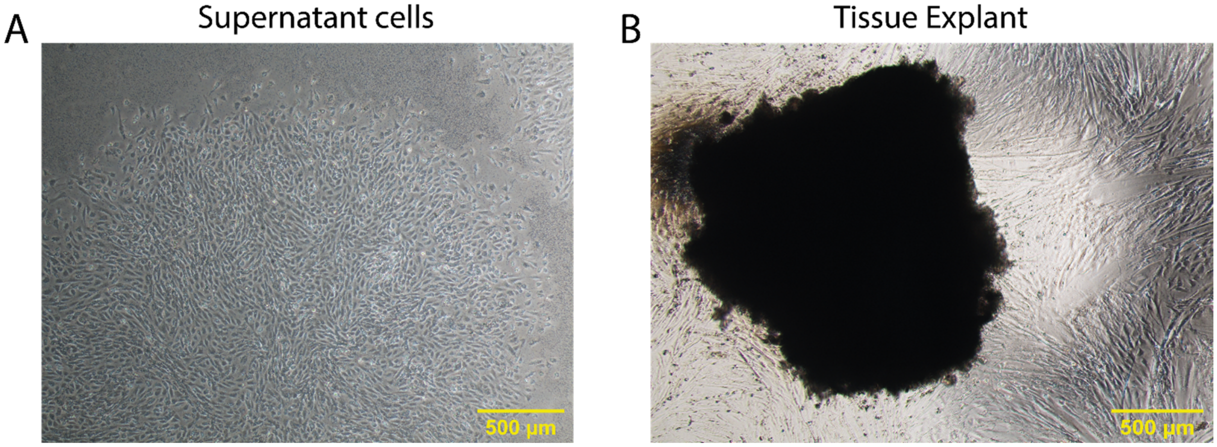

Figure S1: Additional satellite cell material cultivated
*It was possible to achieve more viable cells per gram tissue when cultivating supernatant cells and using tissue explant strategies. **A** show cultivated cells retrieved from the initial supernatant which contained late-attaching cells. **B** is an example of cells expanding from a small piece of tissue, even showing spontaneous fusion.*

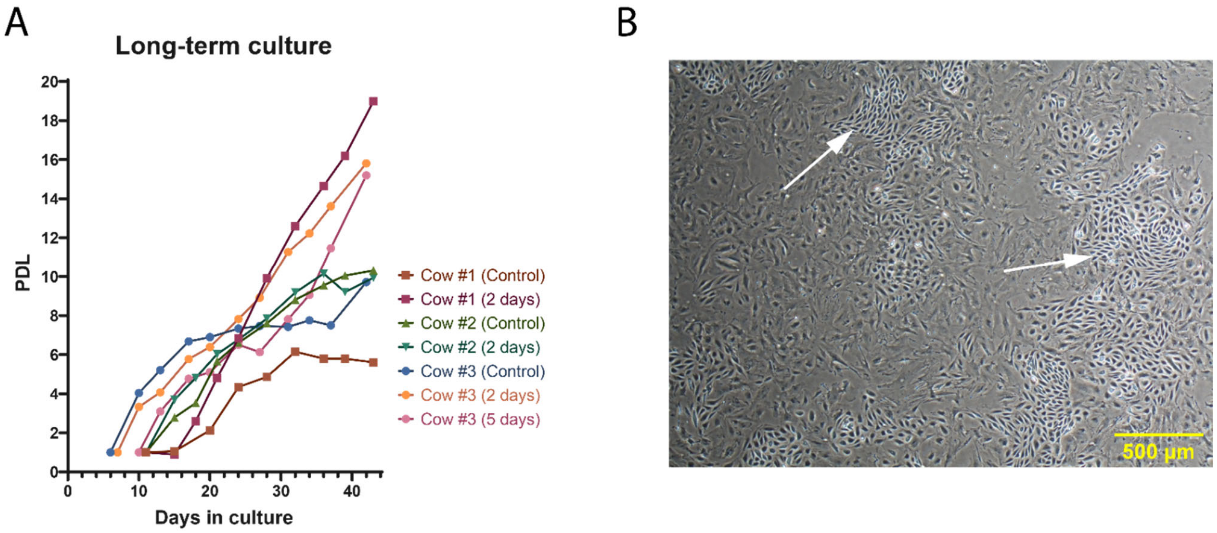

Figure S2: Long-term culture of isolated satellite cells
***A** The population doubling time (PDL) of individual isolates is shown in the 43-day period they were passaged and counted. PDL data points are based on the mean count (2x4 squares) on only one replicate. **B** shows the morphological different cell types (indicated by white arrows) which gradually takes over the cell culture (after at least 20 days) and results in much higher PDLs.*

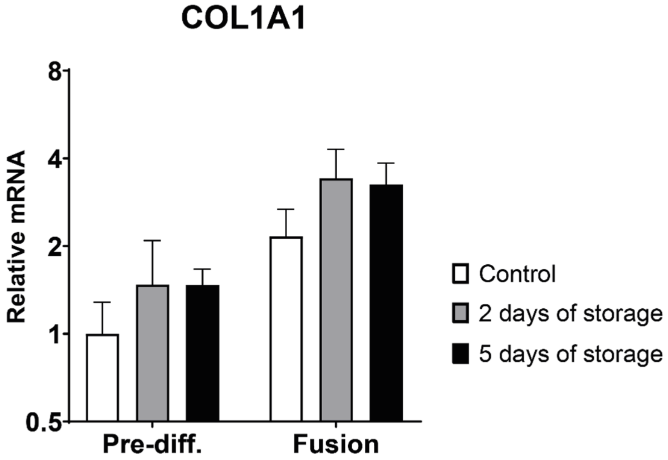

Figure S3: Relative mRNA levels of COL1A1
Relative mRNA expression of the collagen type 1 gene COL1A1 in pre-differentiated (Pre-diff.) satellite cells and after fusion. Data is presented as relative mRNA levels (relative to pre-differentiated control samples). Y-axis is log2 scaled. Data is presented as mean ±SEM based on three cows (n = 3), except 5 days of storage data is only from two cows (n = 2).

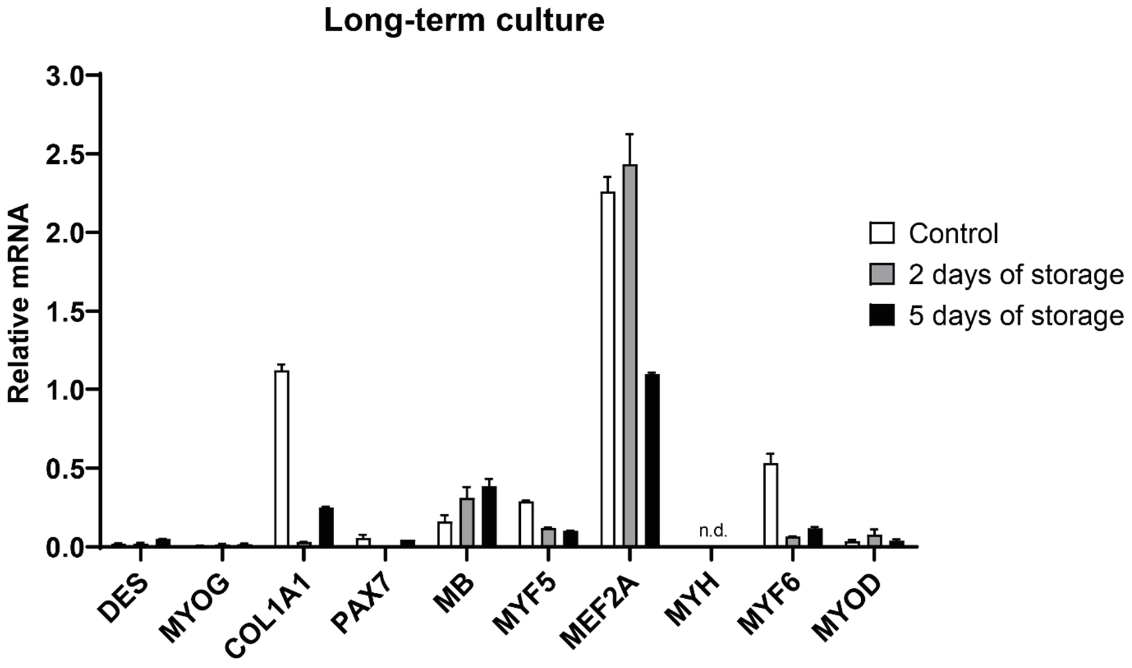

Figure S4: RT-qPCR analysis of long-term culture cells
Relative mRNA expression of all investigated myogenic genes from long-term cultured cells (after 43 days). Data is presented as relative mRNA levels (relative to early phase pre-differentiated control samples). Y-axis is log2 scaled. Data is presented as mean ±SEM based on one cow in technical triplicates (n = 3). N.d. (not detected).
